# Supplementary material for: VE‐Cadherin–Actin Regulation Promotes Mechanotransduction and Monolayer Maturation Involving a Tension‐Sensitive Intermediate State
Source: Adv Sci (Weinh). 2026 Feb 19;13(25):e16423. doi: 10.1002/advs.202516423 (PMC13137838; doi:10.1002/advs.202516423)
Supplement: Supplementary file 1 — Supporting File 1: advs74510‐sup‐0001‐SuppMat.docx; [file ADVS-13-e16423-s001.docx]

**SUPPORTING INFORMATION**

VE-Cadherin–Actin Regulation Promotes Mechanotransduction and Monolayer Maturation Involving a Tension-Sensitive Intermediate State

Jonas Franz^1,2,3#^, Maria Odenthal-Schnittler^1,2,3^, Jan Philip Kipcke^1,2,3^, Jochen Seebach^3^, Zahra Labbaf^5^, Franziska Merten^1,2,3^, Vesna Bojovic^3##^, Muna Taha^3###^ Johannes A. Eble^4^, Erez Raz^1,5^, Milos Galic^6^, Hans Schnittler^1,2,3 *^

^*^ Corresponding author: Hans Schnittler; [hans.schnittler@uni-muenster.de](mailto:hans.schnittler@uni-muenster.de)

**Supplementary Figure 1:**

(**a**) Representative example of phase-contrast time-lapse recordings during cell growth to determine cell density-dependent dynamics. Images were taken at the same location in a growing HUVEC cell culture (scale bar: 100 µm). The cells were initially seeded at a density of 1 x 10^4^ cells/cm^2^. Images are shown at t = 0 hours, t = 60 hours, and t= 120 hours. The cell density at each respective time point is indicated. (**b**) The scheme illustrates the principle of the one-drop assay, which enables the analysis of different cell densities within the same cell culture to investigate G-actin levels (created with Adobe Illustrator 2024 (Adobe Inc., CA, USA)). For this technique, a drop of cell suspension is placed at the center of a cross-linked gelatin-coated glass-bottom chamber. Due to the surface tension of the culture fluid, cells primarily assemble in the center, with decreasing cell numbers settling towards the periphery. Cultures displayed highly confluent (11-12 x 10^4^ cells/cm^2^), confluent (9-10 x 10^4^ cells/cm^2^), and sub-confluent (7-8 x 10^4^ cells/cm^2^) areas. Laser scanning microscopy (LSM) was used to examine the G-actin level with fluorescently labelled deoxyribonuclease I (DNase I) according to ^57^. VE-cadherin labelling served to identify cell borders, and filamentous actin was visualized with phalloidin-TRITC (overview: scale bar 50 µm; cropped areas: scale bar 20 µm). DNase I staining was performed as described. (**c**) LSM images of VE-cadherin-EGFP-overexpressing HUVECs and control cells, immunolabelled as indicated (scale bar: 20 µm). Arrowheads highlight prominent junctional actin and strong junctional integrin β1 recruitment in VE-cadherin-overexpressing cells. (**d**) Left panel (overview): Phase-contrast microscopy of VE-cadherin-EGFP-overexpressing HUVECs and control cells (scale bar: 100 µm). Middle panel: In the cropped area, arrowheads indicate enhanced contrast at cell borders in VE-cadherin-overexpressing HUVECs (scale bar: 50 µm), while controls did not show this phenomenon. Right panel: LSM overview of phalloidin-TRITC-labelled HUVECs, either with VE-cadherin overexpression or EGFP expression. Prominent junctional actin formation (arrowheads) is seen in VE-cadherin-EGFP-overexpressing cells, while stress fibers are largely absent (scale bar: 100 µm).

**Supplementary Figure 2**

(**a**) Flowchart illustrating the measurement of fluorescence intensities at cell junctions using the CellBorderTracker (CBT) as described previously ^66^. CBT was used to acquire a cell border mask based on junction-localized anti-VE-cadherin staining. Next, a distance map was generated using Fiji ImageJ to define the region of interest (ROI), followed by relative actin intensity measurements. (Cropped area) The ROI was defined by extending 5 pixels on both sides of the segmentation line indicated by the white arrows. The relative junctional actin concentration was calculated as the integrated phalloidin-TRITC intensity within the ROI divided by the total cell border length of the ROI. (**b**) LSM images of confluent HUVECs subjected to 18 dyn/cm^2^ U-FSS for t = 0 min, t = 20 min, and t = 240 min, labelled for phalloidin-TRITC, VE-cadherin, and integrin α_V_β3 (scale bar: 20 µm). After t = 20 min of shear stress, a prominent junctional actin ring is formed, accompanied by integrin α_V_β3 recruitment. (**c**) Cropped areas 1 and 2 after t = 20 min of shear stress showing junctional actin and integrin α_V_β3 recruitment to the junctions, as verified by total internal reflection fluorescence microscopy (TIRF) (scale bar: 10 µm).

**Supplementary Figure 3**

LSM and SIM images of confluent HUVECs immunolabeled as indicated. **Scale bar: 20 µm. Control cells (upper panel) and cells after treatment with ROCK inhibitor Y27632 (10µM) for 20 min (second panel). Note the heterogeneous VE-cadherin and actin distribution with JAIL formation and the linearly organized VE-cadherin pattern associated with junctional actin and vinculin (arrowheads) after ROCK inhibition. (Third panel) Exposure to t= 20 min of shear stress (U-FSS) induced VE-cadherin linearization accompanied by the formation of prominent junctional actin and vinculin recruitment. (Fourth panel) Combined effects of U-FSS and Y27632 on HUVEC cultures: cells were preincubated with the ROCK inhibitor for 10 min and subsequently exposed to U-FSS for 20 min. Note the homogeneous linear VE-cadherin pattern with prominent colocalization of actin and vinculin (arrows). (Fifth panel) Cells exposed to U-FSS for 240 min displaying loss of junctional actin and vinculin.**

**Supplementary Figure 4:**

**VE-cadherin-EGFP virus titration and LifeAct-mCherry** **expression.**

(**a**) Merged Western blot using anti-EGFP and anti-VE-cadherin antibodies. Samples were probed with different amounts of VE-cadherin-EGFP lentiviral mix for titration as indicated. α-Tubulin served as an internal loading control. (**b**) HUVECs expressing VE-cadherin-EGFP were stained with anti-VE-cadherin antibody and DAPI, (scale bar: 20 µm). VE-cadherin-EGFP colocalizes with the endogenous VE-cadherin pattern (cropped area). (**c**) HUVECs expressing LifeAct-mCherry were labelled with phalloidin-647, (scale bar: 20 µm).

**Supplementary Figure 5**

Laser scanning microscopy images of confluent HUVECs subjected to 18 dyn/cm^2^ U-FSS for t = 20 min and stained for filamentous actin (phalloidin-TRITC), VE-cadherin, and myosin IIa to demonstrate the presence of the contractile machinery at junctional VE-cadherin (scale bar: 20 µm). (Cropped area 1, scale bar: 10 µm) Arrows indicate the colocalization of myosin IIa and junctional actin.

**Supplementary Figure 6**

Laser scanning microscopy images of confluent HUVECs under static control conditions and after U-FSS (18 dyn/cm^2^) for 20 min, as well as after pre-incubation with Y27632 for 10 min combined with shear stress application, as indicated (scale bar: 20 µm). Cells were immunolabelled for actin filaments with phalloidin-TRITC, VE-cadherin, and integrin β1, as indicated. Y27632 treatment induced a heterogeneous VE-cadherin and actin distribution with both JAIL formation and a linearly organized VE-cadherin pattern associated with junctional actin formation. Cells pre-incubated with Y27632 and subsequently exposed to U-FSS for 20 min show a linear VE-cadherin pattern with prominent actin and integrin β1 overlap.

**Supplementary Figure 7**

(**a**) Laser scanning microscopy images of subconfluent HUVECs co-expressing EPLIN-α-mCherry and EPLIN-β-EGFP after treatment with DMSO, the Rac inhibitor EHT1864 (20 µM, t= 5 min), or the ROCK inhibitor Y27632 (10 µM, t= 20 min). Scale bar, 20 µm. Cells were additionally stained for filamentous actin using phalloidin-647. Note that Rac inhibition (EHT 1864) rapidly suppressed JAIL formation and enhanced stress fiber formation (arrows), whereas ROCK inhibition (Y27632) promoted JAIL formation (arrowheads) and reduced stress fibers. (**b**) Time-lapse recordings of confluent HUVECs expressing LifeAct-mCherry following treatment with a Rho activator (0.25 U/mL). Scale bar, 20 µm; time scale: mm:ss. Prior to Rho activation, cells displayed prominent junctional actin, while Rho activation rapidly induced loss of junctional actin leading to stress fiber formation.

**Supplementary Figure 8**

Laser scanning microscopy overview images (2x2 tiles, 40x magnification, 425 µm x 425 µm) of confluent HUVECs showing filamentous actin (phalloidin-TRITC) and VE-cadherin staining under different U-FSS profiles (scale bar: 50 µm). The white triangle indicates the direction of the U-FSS from the left. (**Image 1**) Control.

(**Image 2**) HUVEC cultures after shear stress of 6 dyn/cm^2^ for 6 h. Note the linear VE-cadherin pattern (arrowheads) accompanied by a prominent junctional actin ring (arrowheads), while stress fibers are largely absent.

(**Image 3**) After upregulation of the shear stress to 36 dyn/cm^2^ in shear stress-adapted cells, stress fibers re-emerge, accompanied by increased cell elongation.

(**Image 4**) After approximately 2-3 h at 36 dyn/cm^2^, cells regain a more cobblestone-like morphology, characterized again by a linear VE-cadherin pattern and prominent junctional actin formation, which is associated with the almost complete disappearance of stress fibers.

(**Image 5**) With continued shear stress at 36 dyn/cm^2^ U-FSS, cells develop actin stress fibers and align and elongate, driven by JAIL formation.

(**Image 6**) Cells subjected to 6 dyn/cm^2^ for 12 hours also adapt over time, showing stress fibre formation and a heterogeneous VE-cadherin pattern, eventually leading to alignment and cell elongation.

**Supplementary Figure 9. Original images of blots found in this study.**

(**a**) Merged Western blot using anti-EGFP and anti-VE-cadherin antibodies. Samples were probed with different amounts of VE-cadherin-EGFP lentiviral mix for titration as indicated. α-Tubulin served as an internal loading control (related to Supplementary Figure 4a). (**b**) Western blot of integrin β1 from confluent HUVECs exposed to 18 dyn/cm^2^ U-FSS as indicated. α-Tubulin served as internal loading control (related to Figure 3f). (**c**) Density-dependent analysis of total actin by Western blot in subconfluent and confluent cells as indicated. Different amounts of total cellular protein (5, 10, 20, 40μg) were probed with an anti-Pan-actin antibody. α-Tubulin served as a loading control (related to Figure 1h). (**d**) Western blot of phospho (ser19)-myosin II light chain (P-ser-MII-LC) levels from confluent HUVECs exposed to 18 dyn/cm^2^ U-FSS as indicated. α-Tubulin served as internal loading control (related to Figure 5c).
